# Comparing Nanohydroxyapatite Graft and Other Bone Grafts in the Repair of Periodontal Infrabony Lesions: A Systematic Review and Meta-Analysis

**DOI:** 10.3390/ijms222112021

**Published:** 2021-11-06

**Authors:** Muhammad Saad Shaikh, Muhammad Sohail Zafar, Ahmad Alnazzawi

**Affiliations:** 1 Department of Oral Biology, Sindh Institute of Oral Health Sciences, Jinnah Sindh Medical University, Karachi 75510, Pakistan; drsaadtanvir@gmail.com; 2 Department of Restorative Dentistry, College of Dentistry, Taibah University, Al Madinah 41311, Al Munawwarah, Saudi Arabia; 3 Department of Dental Materials, Islamic International Dental College, Riphah International University, Islamabad 44000, Pakistan; 4 Department of Substitutive Dental Sciences, College of Dentistry, Taibah University, Al Madinah 41311, Al Munawwarah, Saudi Arabia; alnazzawi@gmail.com

**Keywords:** nanohydroxyapatite, bone grafts, periodontal regeneration, infrabony defects

## Abstract

Objective: To compare the results of periodontal infrabony lesions treated using nanohydroxyapatite (NcHA) graft with other bone grafts (BGs). Methods: Four electronic databases were searched including PubMed (NLM), Embase (Ovid), Medline, and Dentistry and Oral Sciences (EBSCO). The inclusion criteria included randomised controlled clinical trials (RCTs) and controlled clinical trials (CCTs). The clinical results of NcHA were compared with other BGs. For clinical attachment level (CAL) gain, probing pocket depth (PPD) decrease, and gingival recession (REC) change, weighted averages and forest plots were computed. Results: Seven RCTs fulfilled the selection criteria that were included. When NcHA was compared to other BGs, no clinically significant differences were found in terms of each outcome assessed, except the REC change for synthetic BGs as compared to NcHA. Conclusions: The use of an NcHA graft showed equivalent results compared to other types of BGs. To further validate these findings, future studies are required to compare the NcHA and various BGs over longer time periods and in furcation deficiencies.

## 1. Introduction

Periodontitis is a localised inflammatory response to periodontal pocket infection caused by the build-up of opportunistic bacteria and subgingival plaque in the oral cavity [1]. Changes in clinical attachment level (CAL) and probing pocket depth (PPD) are clinical symptoms of loss of support within the periodontium, whereas alveolar bone loss is a radiographic sign. As a result, large periodontal pockets caused by infrabony abnormalities have been identified as an anatomical sequela to periodontal disease (PD) [2]. The vertical, horizontal, and osseous craters have been identified as distinct patterns of alveolar bone loss in PDs [3]. However, the healing and prognosis of infrabony defects after therapy are dependent on a number of parameters, notably the defect shape, which has been reported to impact the availability of cellular and vascular components necessary for regeneration of such defects [4]. The fundamental goal of treating PD has always been to reduce bleeding on probing and inflammation, as well as to reduce PPD and acquire CAL to prevent future attachment loss. Nonetheless, wherever possible, periodontal regeneration has been the ultimate objective in order to promote long-term stability of periodontal tissues [5]. Because conventional surgical methods have limited promise in periodontal regeneration, several forms of bone grafts (BGs) have been widely employed to stimulate bone production and periodontal regeneration [6].

There are wide variations in CAL gain among the different types of biomaterials used. Due to such heterogeneity in outcomes, there is no clear consensus for using specific graft biomaterials. Autogenous bone is currently regarded as the gold standard [7].However, autogenous bone is scarce, and increases morbidity. Because of these concerns, there has been a surge in research on alternate BG materials. These substances are biocompatible, non-antigenic, and non-infectious. Though most are not thought to be osteoinductive, they should at the very least be osteoconductive [8].

Hydroxyapatite (HA) has long been used as a bone substitute material in artificial BGs [9]. Because of its structural and compositional similarity to real mineralised bone, it has been utilised for bone regeneration. HA-based BGs may create a chemical bond with the bone, enhancing bonding characteristics of HA and bone matrix [10,11]. A synthetic nanohydroxyapatite (NcHA) bone replacement material has been successfully introduced for the adjunctive treatment of space-maintaining bone defects following defect fractures and cystectomies, with no discernible side effects [12]. Because of its enhanced osseointegrative properties, NcHA distinguishes a large class of BGs. Schnettler et al. (2004) discovered that NcHA stimulates osteoblast activity, resulting in the production of new bone, and establishes a tight connection with freshly formed bone [13]. Based on the chemical makeup, NcHA material differs from microcrystalline HA biomaterials. Furthermore, the chemical makeup of NcHA is similar to that of bone minerals. Natural bone has a chemical composition of [Ca_10_(PO_4_)_6_(OH)_2_] and a calcium-to-phosphate ratio of 1.67 [14]. Furthermore, the nano-sized particles provide NcHA-unique features, such as the existence of a hydrated surface layer, a high surface-to-volume ratio, and the fact that they are non-apatitic in nature. Because of its propensity for ionic exchange and adsorption, this hydrated layer may contribute to macromolecular interaction. Essentially, it is thought that this layer in the nano-bone mineral, in addition to other strategies engaged in osteogenesis control, is actively involved in the homeostasis process [15,16].

An extensive literature has demonstrated that NcHA can represent a promising class of BGs by demonstrating its effectiveness on periodontal epithelium [17], as well as its role in differentiation and proliferation of periodontal ligament cells, including fibroblasts [18] and osteoblasts [19,20]. Furthermore, NcHA has been shown to play a role in bone regeneration [21], macrophage activity [22], growth factor release [23], angiogenesis [24], and resorbability [23,25], signifying its regenerative potential. Having stated that, NcHA has been utilised as a BG in the regeneration of periodontal tissues, for which there are very little clinical data. As a result, the purpose of this systematic review is to evaluate the clinical performance of NcHA in the regeneration of infrabony defects against other BGs.

## 2. Materials and Methods

This systematic review was created in accordance with the most recent PRISMA (preferred reporting items for systematic reviews and meta-analysis) criteria [26].

### 2.1. Focused Question

The following focused question was developed using the population intervention control outcome (PICO) framework:

*“Does the use of nanohydroxyapatite bone graft provide better clinical results than those of other bone grafts in patients with infrabony defects?”*

The PICO was developed as follows:

Population/problem (P): Patients with infrabony defects.

Intervention (I): Treatment with NcHA BG.

Comparison (C): Treatment with other BGs except NcHA.

Outcomes (O): CAL gain, PPD reduction (primary outcomes), and gingival recession (REC) change (secondary outcome).

### 2.2. Information Sources and Search Strategy

Using PubMed (National Library of Medicine), Medline (EBSCO), Embase (Ovid), and Dentistry and Oral Sciences (EBSCO) databases, a literature search was conducted till February 2021. For the search procedure, the following free-text keywords with Boolean operators were used:

*((“intrabony defects” OR “intra-bony defects” OR “infrabony defects” OR “infra-bony defects”) AND (“nanocrystalline hydroxyapatite” OR “nano-crystalline hydroxyapatite” OR “nanohydroxyapatite” OR “nano-sized hydroxyapatite”)) AND (“open flap debridement” OR “periodontal surgery” OR “access flap surgery” OR “bone grafts” OR “bone replacement grafts” OR “bone substitutes”) AND (“clinical attachment level” OR “probing pocket depth” OR “gingival recession”).*

Furthermore, the reference list of relevant publications was manually searched for further research. Periodontology specialist journals such as *Periodontology 2000*, *Journal of Clinical Periodontology*, *Journal of Periodontology*, and *Journal of Periodontal Research* were also searched. In cases where data were ambiguous or missing, the authors were contacted personally.

### 2.3. Eligibility Criteria

#### 2.3.1. Inclusion Criteria

- Randomised controlled clinical trials (RCTs) or controlled clinical trials (CCTs) comparing NcHA to other BGs.
- Defects with CAL of ≥3 mm, PPD of ≥5 mm, and/or infrabony defect depth of ≥2 mm.
- Studies with a mean follow-up period of at least 6 months or more.
- Studies in English language and conducted on humans.

#### 2.3.2. Exclusion Criteria

- RCTs or CCTs in which NcHA is compared to open flap debridement (OFD) alone or other regenerative treatment except BGs.
- RCTs or CCTs in which NcHA is combined with any other treatment.
- Studies on supra-osseous (horizontal) and furcation defects.
- Studies reporting histological data, conducted on animals and in vitro.
- Case series, case reports, narrative reviews, and systematic reviews.

### 2.4. Literature Screening and Data Extraction

Two independent researchers (M.S.S. and M.S.Z.) conducted a three-stage systematic screening of the retrieved papers. In the first stage, researchers independently examined the retrieved titles and keywords to determine if the inclusion requirements were met. The abstracts were rigorously vetted for relevance to the study issue in the second step. The full text of the screened publications was obtained and thoroughly analysed according to the eligibility criteria in the third step. The primary and secondary outcome measures were examined in all of the included studies. The kappa coefficient (*k*) was used to examine the researchers’ inter-rater agreement [27]. In the event of a disagreement, consent was acquired through discussion with a third reviewer. At least two review writers extracted data independently. Any disagreements were discussed, and a third author was consulted as needed. The following information was collected for each included study:

- Author and year of study.
- Study design.
- Interventional/experimental groups.
- Type of BG used.
- Follow-up period.
- Use of antibiotics in the study.
- Defect characteristics (type and number) and type of tooth.
- Patient characteristics including total number of patients, age range, mean age, gender distribution, smoking history, and drop-outs.
- Primary (CAL gain and PPD reduction) and secondary (REC change) outcomes.

### 2.5. Data Synthesis

The outcomes were calculated using mean differences with standard deviations (mean ± SD) and 95% confidence intervals (95% CI). Review Manager 5.4 (Cochrane Collaboration, Oxford, United Kingdom) for MacOS software was used to conduct meta-analyses, which included papers with similar comparisons and reporting the same outcome measures. For continuous data, mean differences were merged using random-effects models (DerSimonian–Laird’s test) [28]. A *p* value of < 0.05 was judged statistically significant. To prevent inconsistency across research, data from studies with a 6 months follow-up duration were chosen for meta-analysis. The Cochran Q test (significant at *p* < 0.10) for heterogeneity [29] and the I^2^ statistic (25% (low), 50% (moderate), and 75% (high)) [30] were used to assess the significance of any discrepancies in the estimates of treatment effects from different trials. The forest plot was used to depict the weighted mean of the outcome in each study, as well as the final estimate.

For studies with missing data, the SD for the mean change was either (1) imputed from the available data, assuming a correlation coefficient of 0.8 between the baseline and post-intervention SD values, or (2) calculated using statistics that allow for SD calculation, such as CI, standard errors, t-values, *p* values, or F values. Where the above-mentioned techniques did not allow for the determination of the SD for the mean change, the SDs for those studies were imputed from other studies with accessible data in the same meta-analysis [31]. Meta-analyses with fewer than ten research papers were not examined due to the lack of power to identify publication bias; otherwise, funnel plots were employed to assess publication bias [32,33].

### 2.6. Risk of Bias Assessment

The risk of bias was assessed by two review authors (M.S.S. and M.S.Z.). To assess the risk of bias in RCTs, the updated Cochrane risk of bias instrument for randomised trials (RoB2) [34] was employed. The randomisation procedure, variations from the intended interventions, missing outcome data, assessment of the outcomes, and selection of the reported results were all examined in the studies. A study was classified as either “low risk”, when the five domains were judged to be low risk; “some concerns”, when it raised some concerns in at least one domain; and “high risk”, when it was judged to be at high risk in at least one area or when it raised some concerns in multiple domains, significantly lowering confidence in the results.

## 3. Results

**Figure 1:**
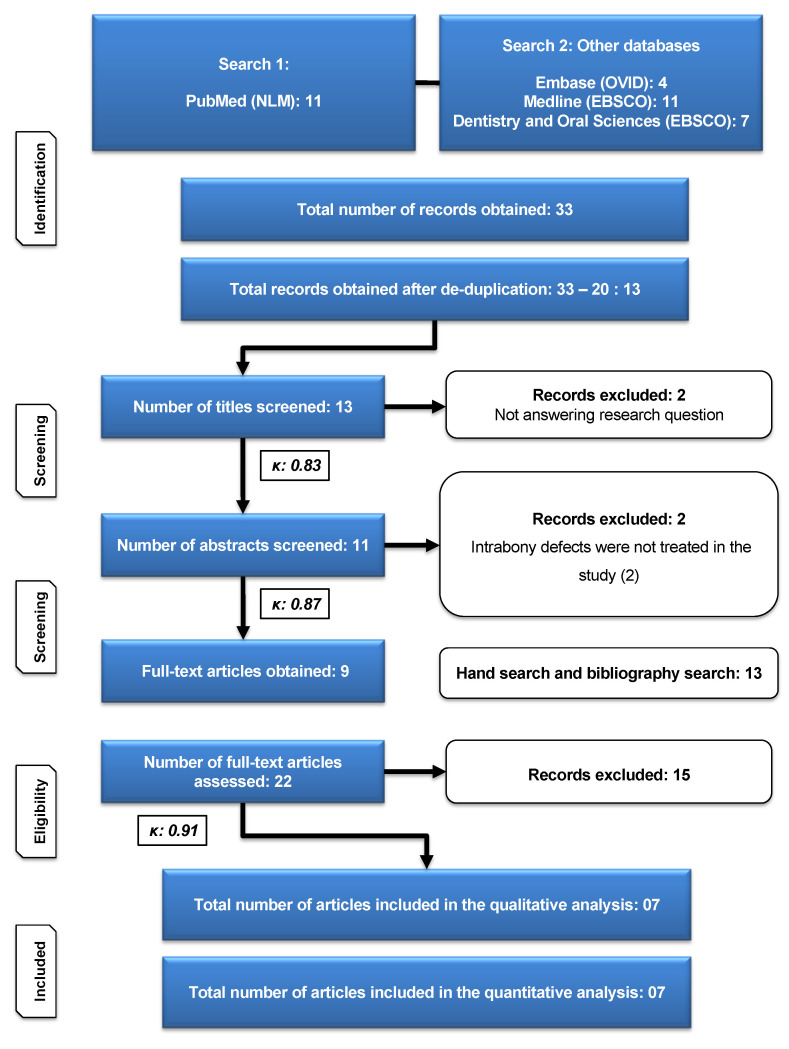
Flow chart according to updated PRISMA guidelines [26].

A search of electronic databases yielded a total of 30 publications. Following the removal of duplicates, 13 documents were obtained. Following the screening of titles, 11 abstracts were chosen. A careful review of the abstracts resulted in the inclusion of nine papers for full-text evaluation. In addition, 13 articles were added as a consequence of manual searching, bringing the total number of articles for full-text review to 22. The examination of 22 full-text publications resulted in the selection of seven studies that met the inclusion criteria for qualitative and quantitative analysis (Figure 1). Following a review of the abstracts, two papers were eliminated, and 15 papers were eliminated after full-text examination (Table 1). The calculated Cohen’s Kappa statistic was 0.83, 0.87, and 0.91 after screening of titles, abstracts, and full texts, respectively, which suggested strong to almost perfect agreement between the two authors based on the commonly cited scale for Kappa statistic interpretation [27].

### 3.1. Characteristics of Included Studies

Table 2 displays descriptive statistics for the seven studies that were included. RCTs were used in all of the included investigations. Except for one study, which used a parallel study design [52], each experiment used a split-mouth design. Five out of seven studies used synthetic BGs [52,53,54,55,56], whereas one study used autogenous BG [57] and another study used xenogenic graft [58]. The maximum follow-up time varied among the included trials, ranging from 6 months in five trials to 9 and 12 months in the other two, respectively [52]. Four studies reported using post-operative antibiotics [52,55,57,58], whereas three studies did not disclose the use of post-operative antibiotics [53,54,56].

### 3.2. Characteristics of Defect and Participant

The investigations found a total of 156 infrabony faults with various morphologies. Two- and three-wall infrabony defects were treated in four articles, while data on defect morphology were unavailable in three others. The position of the teeth was not specified in any of the studies (Table 3). The trials included a total of 86 individuals ranging in age from 20 to 55 years. Gender distribution was addressed in three studies, but not in the other four. Six studies omitted smokers, while one study did not indicate whether smokers were included or excluded. There were no drop-outs in any of the studies, with the exception of one [58], which included two patients who were lost to follow-up (Table 3).

### 3.3. Risk of Bias Assessment

**Figure 2:**
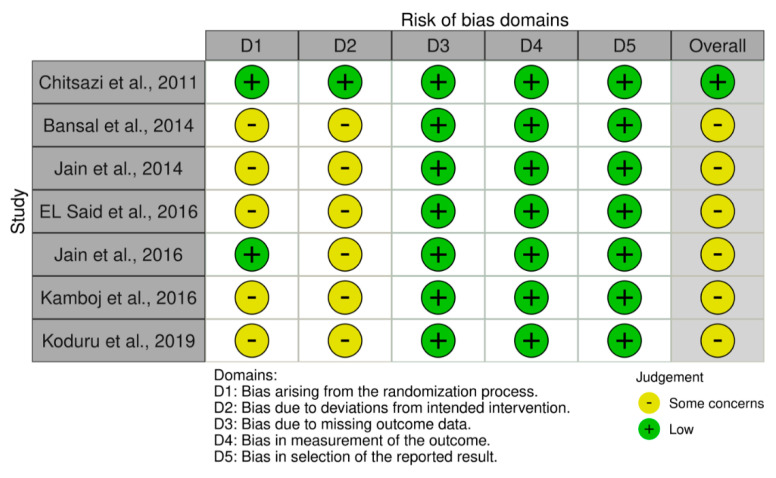
Bias assessment: RoB2 tool for included RCTs.

The new Cochrane RoB2 tool for randomised trials is shown in Figure 2. Six studies were assessed as having “some concerns” about bias, whereas one study was classified as having a “low risk” of bias.

### 3.4. Meta-Analysis

**Figure 3:**
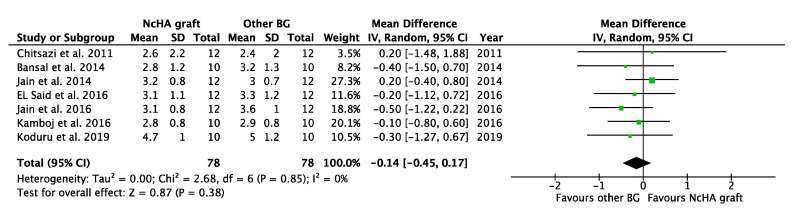
Forest plots demonstrating the comparison of NcHA graft versus other BG (CAL gain). NcHA: nanohydroxyapatite; BGs: bone graft; IV: inverse variance; SD: standard deviation; Total: number of patients; 95% CI: confidence interval.

**Figure 4:**
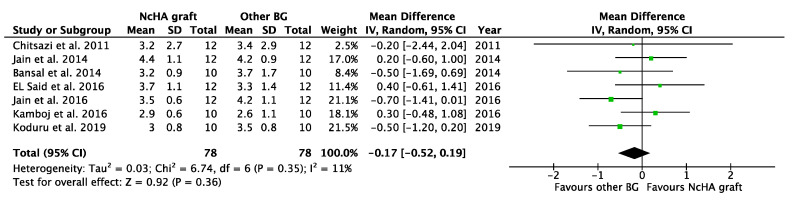
Forest plots demonstrating the comparison of NcHA graft versus other BG (PPD reduction). NcHA: nanohydroxyapatite; BGs: bone graft; IV: inverse variance; SD: standard deviation; Total: number of patients; 95% CI: confidence interval.

**Figure 5:**
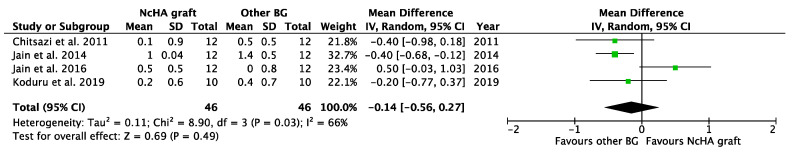
Forest plots demonstrating the comparison of NcHA graft versus other BG (REC change). NcHA: nanohydroxyapatite; BGs: bone graft; IV: inverse variance; SD: standard deviation; Total: number of patients; 95% CI: confidence interval.

Table 4 illustrates the intervention’s effect. All three forest plots (CAL gain, PPD reduction, and REC increase) showed no beneficial effects of NcHA in treating infrabony defects in comparison to other BGs, with a mean difference of −0.14 mm (*p* = 0.38; 95% CI: −0.45–0.17), −0.17 mm (*p* = 0.36; 95% CI: −0.52–0.19) and −0.14 mm (*p* = 0.49; 95% CI: −0.56–0.27), respectively. No heterogeneity was found in CAL gain (Figure 3) analysis (I^2^: 0%), whereas the analysis of PPD reduction (Figure 4) and REC increase (Figure 5) showed low (I^2^: 11%) and moderate heterogeneity (I^2^: 66%), respectively. Seven trials were eligible for CAL gain and PPD reduction meta-analysis, whereas four trials were included in the analysis for REC increase.

### 3.5. Subgroup Analysis

**Figure 6:**
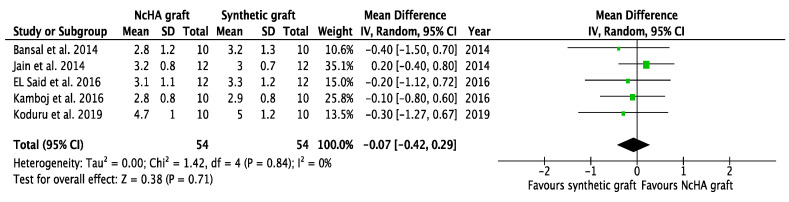
Forest plots demonstrating the comparison of NcHA graft versus synthetic graft (CAL gain). NcHA: nanohydroxyapatite; IV: inverse variance; SD: standard deviation; Total: number of patients; 95% CI: confidence interval.

**Figure 7:**
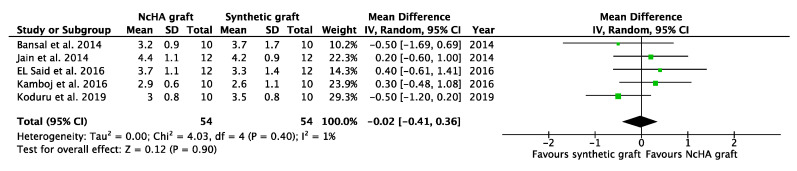
Forest plots demonstrating the comparison of NcHA graft versus synthetic graft (PPD reduction). NcHA: nanohydroxyapatite IV: inverse variance; SD: standard deviation; Total: number of patients; 95% CI: confidence interval.

**Figure 8:**
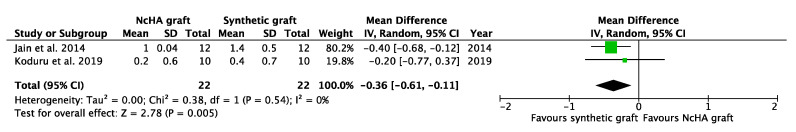
Forest plots demonstrating the comparison of NcHA graft versus synthetic graft (REC change). NcHA: nanohydroxyapatite; IV: inverse variance; SD: standard deviation; Total: number of patients; 95% CI: confidence interval.

Based on the type of BG, a subgroup analysis was conducted, which was only possible for the NcHA graft versus synthetic BGs. All three analyses in the subgroup showed statistically insignificant differences (*p* > 0.05) between NcHA graft and synthetic BGs except REC increase (*p* < 0.05). Mean differences for CAL gain (Figure 6), PPD reduction (Figure 7), and REC change (Figure 8) was −0.07 mm (*p* = 0.71; 95% CI: −0.42–0.29), −0.02 mm (*p* = 0.90; 95% CI: −0.41–0.36) and −0.36 mm (*p* = 0.005; 95% CI: −0.61–0.11), respectively. Heterogeneity was found to be negligible for CAL gain (similar to the previous meta-analysis) (I^2^: 0%) and PPD reduction (reduced from I^2^: 11% to I^2^: 1%) analyses, whereas for the REC change, no heterogeneity was found (I^2^: 0%).

## 4. Discussion

Better knowledge of periodontal disease aetiology and tissue responses to various surgical methods has resulted in significant advances in periodontal therapy. In the realm of periodontal regeneration, innovative therapies and adjuncts to established treatment methods are constantly being researched and presented [59]. Several treatment approaches for periodontal regeneration have been utilised, including barrier membranes [60,61], BGs [62,63], EMD [64,65,66], and growth factors [67]. In humans, there is considerable histological evidence of BGs, indicating periodontal unit regeneration with new cementum, alveolar bone, and a functioning periodontal ligament [68]. Since the introduction of nanotechnology, many materials have been created for the treatment of bone deformities, with promising outcomes.

The current study thoroughly compared the clinical efficacy of NcHA with standard periodontal therapy and alternative regeneration methods for periodontal infrabony lesions. A meta-analysis was performed in this paper to examine changes in CAL, PPD, and REC in infrabony defects. The overall meta-analysis showed no additional benefits of NcHA over other BGs, demonstrating comparable results of CAL gain, PPD reduction and REC increase between both test (NcHA) and control (other BGs) groups. This is in disagreement with a systematic review that demonstrated beneficial effects of a BG (anorganic bovine-derived hydroxyapatite matrix/cell-binding peptide graft) rather than the OFD, in terms of CAL gain and PPD reduction [63]. However, in the subgroup analysis (NcHA versus synthetic BGs), only REC change demonstrated statistically significant results in favour of synthetic BGs, rather than NcHA grafts (*p* < 0.05).

In terms of smoking history, two studies included smokers, three studies did not report on smokers, and eleven studies omitted smokers. As a result, drawing conclusions about smoking as a confounding factor in regenerative treatment using NcHA transplant from the data in this research is difficult. Smoking has been shown to be a significant risk factor for periodontal disease [69]. Similarly, smokers’ reaction to periodontal therapy is less advantageous than non-smokers’ [70], and smokers have lower CAL gains for regeneration treatment as compared to non-smokers. The majority of the research followed comparable post-operative procedures; however, antibiotics were prescribed in four trials [52,55,57,58], and three studies gave no data on any specific post-operative measures [53,54,56]. The benefits of post-operative antibiotics have not been evaluated because the bulk of research utilised post-operative antibiotics. As a result, it is fair to anticipate that the administration of post-operative antibiotics may act as a confounding factor [71,72].

Although the present review study suggests similar results for NcHA against other BGs, more RCTs are needed to adequately assess its role in periodontal regeneration. Specifically, NcHA graft needs to be compared against different types of BGs, as in this review, other BGs were combined. Furthermore, another consideration that can be taken into account is the follow-up time period. Most studies included in this review analysis showed results at 6 to 12 month follow-ups. To evaluate precise effectiveness of NcHA in periodontal infrabony defects, high-quality, long-term studies comparing NcHA graft with conventional periodontal surgery as well other regenerative techniques are required. Apart from that, the role of NcHA graft should be investigated in other periodontal defects, such as furcation involvements.

This systematic review has a few limitations. First, it should be noted that this meta-analysis integrated research results of regenerative surgery performed in defects with diverse morphologies (i.e., one-, two-, and three-walled, and combinations thereof), utilising various types of BGs. Second, the lack of consistency and standardisation may have led to heterogeneity of results in the meta-analysis of PPD reduction and REC increase. Nevertheless, in the subgroup analysis, heterogeneity was considerably reduced. As a result, these findings should be taken with care. Furthermore, due to a paucity of data, no meta-analysis could be conducted on other types of BGs (except synthetic BGs), defect shape, and surgical flap designs, which are well-known factors influencing outcomes following regenerative treatment [65,73,74]. Many of the studies used for the final analysis did not disclose data on the treatment of interdental papilla and primary wound closure during early wound healing. Finally, according to the RoB2 method, the quality evaluation of trials included in the quantitative analysis was mostly classed as “some concerns”, since most studies had some issues in the randomisation procedure and deviations from the planned intervention. Despite a bias assessment, the decision to include each study was ascribed to the fact that some of them were older, and there was a shortage of data in the literature.

## 5. Conclusions

The present systematic review and meta-analysis showed equivalent effects of NcHA graft and other BGs, in terms of CAL gain, PPD decrease, and REC increase. To verify the potential for periodontal regeneration, future trials comparing the NcHA graft to other BGs, with longer follow-ups, and investigating furcation deficiencies are essential.

## Figures and Tables

**Table 1:** Excluded studies with reasons.

Author/Year	Reason for Exclusion
**Reasons for exclusion after abstract screening**	
Schwarz et al., 2008 [35] Schwarz et al., 2009 [36]	Infrabony defects were not treated in the study
**Reasons for exclusion after full text screening**	
Elbattawy and Ahmed (2021) [37] Pietruska et al., 2012 [38] Kasaj et al., 2008 [39]	NcHA compared to OFD only group
Anitha et al., 2017 [40] Verardi et al., 2020 [41]	Case series
Horváth et al., 2013 [42]	No control group
Ghoniem et al., 2016 [43]	NcHA compared to non-surgical treatment
Singh et al., 2012a [44] Singh et al., 2012b [45]	No NcHA-only group
Bahammam and Attia 2021 [46] Yousef et al., 2018 [47] Dayashankar et al., 2017 [48] Elgendy and Shady 2015 [49] Pilloni et al., 2014 [50] Al Machot et al., 2014 [51]	NcHA compared to other regenerative material(s) except BGs

BGs: bone grafts; NcHA: nanohydroxyapatite; OFD: open flap debridement.

**Table 2:** Study characteristics.

**Author and Year**	**Type of Study**	**Type of BG Used**	**Experimental Groups**	**Follow-Up (Months)**	**Antibiotics**
Chitsazi et al., 2011 [57]	Split-mouth design RCT Single-centre Prospective	Autogenous	Test: NcHA + OFD Control: ABG + OFD	6	Y
Bansal et al., 2014 [53]	Split-mouth design RCT Single-centre Prospective	Synthetic	Test: NcHA + OFD Control: HA + OFD	6	NR
Jain et al., 2014 [55]	Split-mouth design RCT Single-centre Prospective	Synthetic	Test: NcHA + OFD Control: ß-TCP + OFD	6	Y
EL Said et al., 2016 [54]	Split-mouth design RCT Single-centre Prospective	Synthetic	Test: NcHA + OFD Control: HA + OFD	6	NR
Jain et al., 2016 [58]	Split-mouth design RCT Single-centre Prospective	Xenogenic	Test: NcHA + OFD Control: DBM + OFD	12	Y
Kamboj et al., 2016 [56]	Split-mouth design RCT Single-centre Prospective	Synthetic	Test: NcHA + OFD Control: HA + OFD	6	NR
Koduru et al., 2019 [52]	Parallel design RCT Single-centre Prospective	Synthetic	Test: NcHA + OFD Control: Bioactive glass synthetic + OFD	9	Y

ABG: autogenous bone graft; ß-TCP: ß-tricalcium phosphate; DBM: demineralised bone matrix; HA: hydroxyapatite; N: no; NcHA: nanohydroxyapatite; NR: not reported; OFD: open flap debridement; RCT: randomised controlled clinical trial; Y: yes.

**Table 3:** Defect and participant characteristics.

Author/Year	Defect Characteristics			Participant Characteristics				
	Defect Type	Number of Defects	Tooth Type	Total Patients	Age Range/Mean Age	Gender (M/F)	Smoking	Drop-Outs
Chitsazi et al., 2011 [57]	2- to 3-wall IBDs	Test: 12 Control: 12	NR	12	NR/38	NR	Ex.	0
Bansal et al., 2014 [53]	NR	Test: 10 Control: 10	NR	10	20–50/NR	8/2	Ex.	0
Jain et al., 2014 [55]	2- to 3-wall IBDs	Test: 12 Control: 12	NR	12	20–50/NR	6/6	Ex.	0
EL Said et al., 2016 [54]	2- to 3-wall IBDs	Test: 12 Control: 12	NR	12	40–55/NR	NR	Ex.	0
Jain et al., 2016 [58]	NR	Test: 12 Control: 12	NR	10	20–45/NR	7/3	Ex.	2
Kamboj et al., 2016 [56]	2- to 3-wall IBDs	Test: 10 Control: 10	NR	10	NR/NR	NR	NR	0
Koduru et al., 2019 [52]	NR	Test: 10 Control: 10	NR	20	25–55/NR	NR	Ex.	0

Ex.: excluded; IBDs: infrabony defects; In.: included; NcHA: nanohydroxyapatite; NR: not reported.

**Table 4:** Changes in CAL, PPD, and REC.

Author/Year	CAL Gain (mm)	PPD Reduction (mm)	REC Change (mm)
Chitsazi et al., 2011 [57]	Test: 2.6 ± 2.2 Control: 2.4 ± 2.0	Test: 3.2 ± 2.7 Control: 3.4 ± 2.9	Test: 0.1 ± 0.9 Control: 0.5 ± 0.5
Bansal et al., 2014 [53]	Test: 2.8 ± 1.2 Control: 3.2 ± 1.3	Test: 3.2 ± 0.9 Control: 3.7 ± 1.7	NR
Jain et al., 2014 [55]	Test: 3.2 ± 0.8 Control: 3.0 ± 0.7	Test: 4.4 ± 1.1 Control: 4.2 ± 0.9	Test: 1.0 ± 0.04 Control: 1.4 ± 0.5
EL Said et al., 2016 [54]	Test: 3.1 ± 1.1 Control: 3.3 ± 1.2	Test: 3.7 ± 1.1 Control: 3.3 ± 1.4	NR
Jain et al., 2016 [58]	Test: 3.1 ± 0.8 Control: 3.6 ± 1.0	Test: 3.5 ± 0.6 Control: 4.2 ± 1.1	Test: 0.5 ± 0.5 Control: 0 ± 0.8
Kamboj et al., 2016 [56]	Test: 2.8 ± 0.8 Control: 2.9 ± 0.8	Test: 2.9 ± 0.6 Control: 2.6 ± 1.1	NR
Koduru et al., 2019 [52]	Test: 4.7 ± 1.0 Control: 5.0 ± 1.2	Test: 3.0 ± 0.8 Control: 3.5 ± 0.8	Test: 0.2 ± 0.6 Control: 0.4 ± 0.7

CAL: clinical attachment level; NR: not reported; PPD: Probing pocket depth; REC: recession.

## Data Availability

Data supporting the reported results can be found at PubMed (National Library of Medicine), Medline (EBSCO), Embase (Ovid), and Dentistry and Oral Sciences (EBSCO) databases.
